# Gold Foil

**Published:** 1877-02

**Authors:** W. Storer How


### GOLD FOIL.

BY DR. W. STORER HOW.

Gentlemen of the Cincinnati Dental Society:—In presenting, by your appointment, the subject of Dental Foils for our resumed consideration this evening, I shall limit my observations and speculations within the range of gold products, and at the very outset can but think how often we have been foiled with foils, and how little is really known about the gold of which we talk, and want, and use so much.

My occupations and hindrances have been barriers in the way of extended and laborious research along the line thus indicated, and so I shall doubtless betray the ignorance which led me to imagine the field so barren by setting forth briefly what I wish I did know on the subject of dental foils with especial relation to gold, in the hope that those who are better informed may be thus led to impart their knowledge in such a way that practical results will generally follow to an extent which will leave no room for the doubts now so freely shuttled from one to another, and college to college, as to whether annealing gold foil makes it "hard" or makes it "soft." There were various speculations among us in the previous discussion as to the so called "hardening" of pellets or ropes or strips of beaten gold foil when heated in the flame of the burner, and it was alleged that the rolled gold does not "harden" under the same treatment, hence the theory was advanced that the radial extension of gold in the process of foliation, confuses the crystals or ultimate particles so that when suddenly called on by the spirit flame they get their backs up right where they happen to be, without any reasonable relation to their neighbors, and thus are very intractable and difficult of management, whereas, the pursuasive embrace of the rollers fosters friendly associations among the atoms, so that they know just where they are when suddenly aroused, and will unresistingly take any place assigned them. Before expressing an opinion on that theory, I should like to know about its fact foundation, and therefore inquire if it is certain that beaten foil and rolled foil of the same or like numbers behave differently after the usual operative annealing?

If I should be told that numerous experiments had established the fact of a marked difference, then I should ask if the two forms were prepared from precisely similar gold. A question based upon my ignorant supposition that there are different kinds of gold. That African gold, and California gold, and Australian gold have probably as marked characteristics as Swedish iron, Russian iron, or American iron.

I have no conception of the quarter from which an answer may come, but I should be most surprised if a foil manufacturer should exhibit an understanding of the question, for I have more than twenty years experience to back me in the assertion that no two books of gold foil from the same maker ever behaved just alike under the points of my pluggers, and in saying this I make due allowance for varying moods in myself and my patients; for atmospheric variations; for gaseous adhesions; for dirt; for dental depot brutalities, and for everything else I can think of except the uniform manufacturing ignorance which has never failed to thwart my best endeavors to get gold of something near constant working qualities. Surely the name of the article is miserably consonant with my feelings when compelled here to declare that I have been thus *foiled* from the first to this day.

Indeed, I may be said to have inherited the disability from my preceptor (Dr. Benj. Lord, of New York) whose like experience long antedates mine. In fact, so far as my observation goes, the profession generally will bear testimony to the certain uncertainty of every maker's gold foil. But perhaps the strongest corroboration is found in the successive announcement of every foil maker, coming down through those years, to the effect that *he* could furnish "pure gold," "soft foil," etc., and yet to this day there is no constant gold foil. Doubtless I shall hear again and again of dentist's being whimsical or notional about foil, which will be solemnly declared to be "absolute gold," and "all out of the very same ingot," as if those were intelligent answers to the incessantly recurring question, "Why did not that last lot of foil work as well as the first?"

In the handling of foil after manufacture, and in transit, no account seems to be taken of the facts that flexion, or purcussion changes the structure of gold, facts of practical moment in laboratory manipulation of gold plate, which requires frequent annealing to restore the disturbed structure, but facts altogether ignored in the much more delicate oral operations.

I believe that many first class failures, i. e. apparently good fillings that have soon come out, were largely due to the hardening process of "booking" the foil, and the shocking practices of dealers and others in throwing the books down and stamping on them. Quite possibly it may be the resilient assertion of its dignity that that springs the gold out of the cavity into which it has been so unwillingly crammed, for beyond all doubt many hitherto neglected or unnoticed causes will be found at the bottom of our common failures.

We have all noticed the disposition or tendency of foil in its so called cohesive condition to shrink upon itself under the plugger, by a sort of centripetal action, which results in a condensed mass of gold that is loose in the cavity, and made yet looser by further superficial efforts to compact the foil, and when such work is done under the mallet, and especially under the rapidly multiplied blows of the machine mallet, we have added to centripetal action the percussive disintegration of structure previously referred to, with the combined production of a gold sand ball, which, if the mouth could be kept quite dry, might almost be heard to rattle in its socket on the occasion of a vêry laughable shake of the head.

In the use of serrated points with hand pressure, the centripetal tendency is reduced to its minimum, both by the lateral displacement of the several wedge points, and by the porous character of the growing plug, which, on account of such porosity admits of a spreading condensation when built into its place, and we may therefore say just here that there is a class of cavities which can be more durably filled with foil in such a porous condition, than with foil more closely compacted to form a dense plug, which however perfect in itself is yet a very imperfect filling. We have all seen foil fillings that were soft to a degree of sponginess, but were nevertheless steadily subserving the prime purpose for which they had been put in position years ago, and would doubtless preserve those teeth for years to come, if happily they should escape the sharp exploring point, directed not with gentle search, along the margins of the cavity, but with almost savage exultation right into the bowels of the plug which forthwith must give place to a hard gold button, polished to a mirror surface, and ready at any moment within the following few months to illustrate the difference between a "soft" country bumpkin faithfully filling his sphere from year to year, and a "hard" flashy youth of loose habits who can not stay long in one place.

Our attention has been diverted by the glare and glitter of brilliant operations, so that very many of us have lost sight of the real uses of dental foils, and have come to consider only how hard fillings can be made under the mallet, rather than how teeth can be saved by plugs that will remain though their integral structures may be theoretically very imperfect. Practically it is of the first and last importance that there shall be continuous contact of the walls of the plug with the walls of the cavity, and practically, the gold foil of commerce as now prepared and used, is not made to accomplish those ends, unless by exceptionally skillful operators, whose delicate manipulations and persistent energy, with thoughtful experience, enables them to overcome the obstacles mentioned, and actually save many teeth with even —— Blank's foil.

The various attempts to supersede foil by other preparations of gold sufficiently attest the general appreciation of foil's defects, and the great want of either a substitute or its improvement, and my own experience leads me to expect more from the latter than the former; hence these ingnorant suggestions and speculations, not to mention the dogmatic spicery thrown in gratis. I therefore merely mention crystal, sponge, plastic and crystalline gold as in their behaviour under the plugger corroborative of what I have said regarding the working properties of our common foils, and in view of what appears to me to be the natural and mechanical inherencies of gold, I do not expect the coming preparation to appear in the so-called crystalline form, although persons of much wider and longer and practical experiences are saguine in their hopes of that result; my own anticipations rather point to first, a foil formed by machine electro plate on a metal, or metallic conductor to be subsequently separated from the foil by acids or otherwise, which will leave the gold molecules or atoms in orderly and peaceful apposition, ready for the most pliable accommodation to their destined incarceration, and carry no concealed spring saws with which to cut their way out of their cells. And second, a foil thus formed on a very thin sheet of tin foil to be used in conjunction with the tin as a "soft" foil, for of course it would not bear "annealing" as commonly practiced, and would have but a limited range of application in suitably situated cavities.

These two imaginary forms of electro gold and electro tin-gold would seem to me to meet all the initial indications for the large class of dental operations outside the range of proper plastic plugs.

With regard to the immediate forms of prepared foil, it is to be said that the shapes should be such as to primarily effect the least flexion or strain of texture in the leaf, and secondarlly afford the greatest facility for placement in the cavity. Obviously, few of the commercial preparations fulfill both of these essential conditions, and the damaged state in which the foil comes to the dentist precludes the possibility of his compliance with such instructions, but the nearest approach he can make so far as I am able to determine is, with the foil shears to bisect a leaf, cut cross strips of a width suitable for the work to be done; hold in the upturned palm of the left hand a whole and clean Barnum, on which to lay smoothly a strip of foil, and with the edge of one shear blade in the right hand, gently press the middle of the strip on the rubber, and by closing the left hand, fold the strip on the blade which is then withdrawn, and the process repeated until the desired width is obtained. In practice a piece of Barnum rubber may be readily stitched to form a mitten convenient for the purpose, and the details of the process should be performed with a full comprehension of both the facts and the fancies hereinbefore set forth concerning this most valuable and peculiar metal.

There has been a recent attempt to manufacture foil strips for market, but those I have seen were so much wider and thicker than those to which I have accustomed myself, that I made no experimental test of the commercial article, although it is my purpose to do so, as in my judgment that is the preferable form, next to which I should rank the coiled cylinder as approximately complying with the indications, and with alacrity commend the bottle package as a much needed protection from the dirts and the vandals.

Superficially considered, the commercial "pellets" would seem to meet measurably the requirements specified, but a careful examination of the extent, the hardness and the disruptedness of the multiplied edges of the lamina, will show the theoretical unfitness of such forms under the limitations herein described, while "blocks" and "shreds" in like manner fall under the same category. Ropes also must be condemned as affecting a torsionary deterioration of the foil, to say nothing of the crimped and crumpled and crushed and finger pinched wads of the average office practice.

In attempting, by the application of heat, to disentangle the snarled atoms, or to rearrange the irate crystals of either the prepared or plain foils after they become soiled, care should be taken to anneal gradually at a low and steadily increasing temperature to give time for the redisposition of the particles, not allowing the metal to pass the low red point and withdrawing through the warm current above the flame.

There is very much more to be said on this exceedingly important subject, but I limit myself to the presentation of such parts of it as have not to my knowledge received due consideration, and in conclusion I summarize them as follows:

First. There are probably different kinds of gold; therefore that found most suitable for dental foil should be selected by the manufacturer.

Second. Care should be taken to obtain the metal in absolute and unvarying purity.

Third. The method of manufacture ought always to recognize the sensitive disposition and composition of gold, in order that the utmost placability may be obtained.

Note.—Talk with a gold beater, or read the writings of one, if you would learn the certainty of gold's most surprising behavior, and the superstitions invoked to account for them, as well as to cover confessed ignorance where knowledge might reasonably be expected.

Fourth. There should be a standard of form with variations of size to meet the principal exigencies of practice, and I have suggested the folded strip as such standard.

Fifth. The package should be such as to afford seclusion from both fluid and solid dirt, and also a protection from either thoughtless or careless disturbance of the foil form until the moment of its placement in its home.

If these considerations and suggestions so imperfectly and incompletely presented shall in any way lead to an advance in the art with reference to which one of its adepts has recently written, "In regard to the manufacture of gold foil, I desire to say that nothing new has been discovered and no art lost for forty years," I shall be amazingly gratified indeed, and rejoice together with you over such a wished for result of our gold speculations.

